# Expression of Nuclear Factor Kappa B and Survivin in Psoriasis

**DOI:** 10.5402/2012/257059

**Published:** 2012-11-25

**Authors:** Kamer Gunduz, Peyker Temiz, Gulsum Gencoglan, Isil Inanir, Arzu Catalkaya

**Affiliations:** ^1^Department of Dermatology, Faculty of Medicine, Celal Bayar University, Manisa, Turkey; ^2^Department of Pathology, Faculty of Medicine, Celal Bayar University, Manisa, Turkey

## Abstract

*Background and Objective*. Suppression of apoptosis has been proposed as a mechanism responsible for epidermal thickness in psoriasis. Survivin is a member of the inhibitor of apoptosis family. Nuclear factor kappa B (NF-**κ**B) is one of the transcriptional factors that regulate many genes affecting apoptosis. The aim of this study was to determine survivin and NF-**κ**B expressions in psoriasis in comparison with normal epidermis. *Patients and Methods*. Immunohistochemical expressions of survivin and NF-**κ**B were investigated in 41 psoriatic and 21 normal skin samples. *Results*. Diffuse nuclear survivin expression in all epidermal layers was seen in all of the psoriatic samples. NF-**κ**B expression in different epidermal locations was seen in all of the psoriatic samples. Nuclear staining was positive in 40 psoriasis samples. Similar survivin and NF-**κ**B expressions were observed in normal skin samples. *Conclusion*. Since similar expressions are seen in both normal and psoriatic epidermis, no important roles for survivin and NF-**κ**B can be attributed in epidermal proliferation and thickness seen in psoriasis.

## 1. Introduction

Psoriasis is characterized by hyperproliferation and abnormal differentiation of epidermal keratinocytes, lymphocyte infiltration consisting mostly of T lymphocytes, and various endothelial vascular changes in the dermal layer, such as angiogenesis, dilation, and high endothelial venule formation. The proliferative cell population is approximately doubled in psoriasis, whereas the cell cycle is more than 8 times shorter (36 versus 311 hours), and daily production of keratinocytes in psoriatic lesions is approximately 28 times greater than that in normal epidermis [1].

Apoptosis represents a counterbalance to proliferation, and decreased apoptosis is generally thought to be associated with epidermal hyperproliferation. Therefore, suppression of apoptosis has been proposed as a mechanism responsible for epidermal thickness in psoriasis [2]. Apoptotic index of the germinative compartment was found to be 0.12% in normal epidermis, 0.035% in established psoriasis, and 0.31% in regressive psoriasis, suggesting that apoptosis may be involved in the regression of psoriatic hyperplasia [3].

Nuclear factor kappa B (NF-*κ*B) plays an important role in balancing growth and differentiation in the epidermis. The ongoing examination of NF-*κ*B signaling has revealed ever expanding knowledge of its role in stress responses, apoptosis, cell survival, oncogenesis, and development [4]. Activation of the NF-*κ*B transcription factor is one of the antiapoptotic mechanisms initiated during keratinocyte differentiation. NF-*κ*B is not activated in proliferating epidermal cells, but it is activated and translocated to the nucleus upon differentiation [5].

Survivin is a bifunctional protein that regulates cell division and suppresses apoptosis [6]. It is described as a member of the inhibitor of apoptosis (IAP) family that is expressed in most human cancers and also known to be a regulator of mitosis. In malignant cells, survivin expression is upregulated during the G2 M phase of the cell cycle and peaks during mitosis [2].

There is limited data about the antiapoptotic roles of survivin and NF-*κ*B in psoriasis. The aim of this study was to determine survivin and NF-*κ*B expressions in psoriasis in comparison with normal epidermis.

## 2. Materials and Methods

### 2.1. Tissue Collection

Skin punch (6 mm) biopsies were obtained from 41 patients who were clinically diagnosed as having psoriasis vulgaris, and 21 normal skin biopsies from healthy volunteers were used as the control group. The Local Research Ethics Committee approved the study protocol. After the diagnosis of psoriasis was confirmed by histopathological examination, further laboratory investigations were performed.

### 2.2. Immunohistochemical Analysis

Five-micrometer-thick sections cut from paraffin embedded blocks were deparaffinized and then stained with survivin (NeoMarkers, Fremont, CA, USA, RB-9245-R7, LOT: 9245R808D) and NF-kappa B/p65 (NeoMarkers, Fremont, CA, USA, RB-1638-R7, LOT: 1638R801C) antibodies by using streptavidin-biotin immune peroxidase method (Ultravision Detection System, Lab Vision, Fremont, CA, USA, REF: TP-125-Hl, LOT: PHL 80807). In the staining procedure, the antigens were extracted by boiling in the pressured container, blocked for 10 minutes, and then incubated for 45 minutes. Diaminobenzidine (DAB) (ScyTec, Logan, Utah, USA, REF: ACK125, LOT: 15827) was used as chromogen. Appropriate positive and negative controls were used. The staining patterns were evaluated by the pathologists with light microscopy.

## 3. Results

### 3.1. Survivin

**Figure 1:**
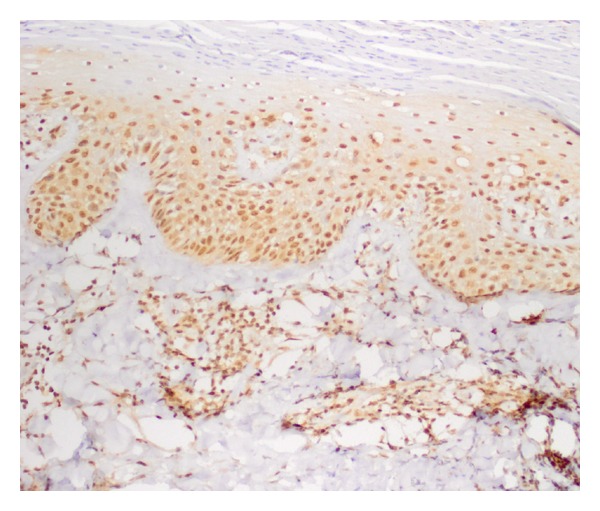
Diffuse nuclear staining with survivin in all layers of psoriatic epidermis (×200).

**Figure 2:**
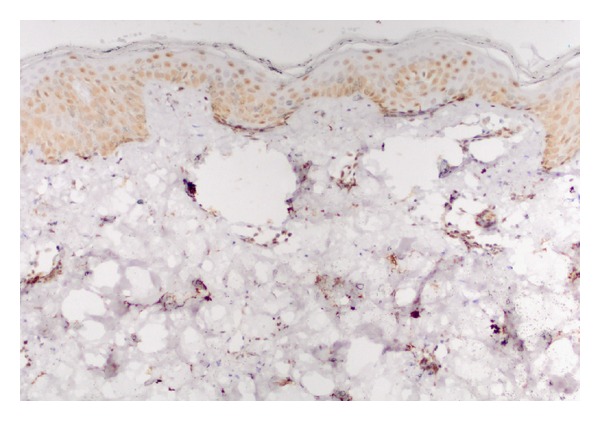
Nuclear staining with survivin in normal epidermis (×200).

Staining properties of survivin are summarized in Table 1. Forty out of 41 psoriatic lesions showed diffuse nuclear positivity. The staining was both nuclear and cytoplasmic in one sample (Figure 1). Normal skin samples showed diffuse nuclear staining (Figure 2).

### 3.2. Nuclear Factor Kappa B

**Figure 3:**
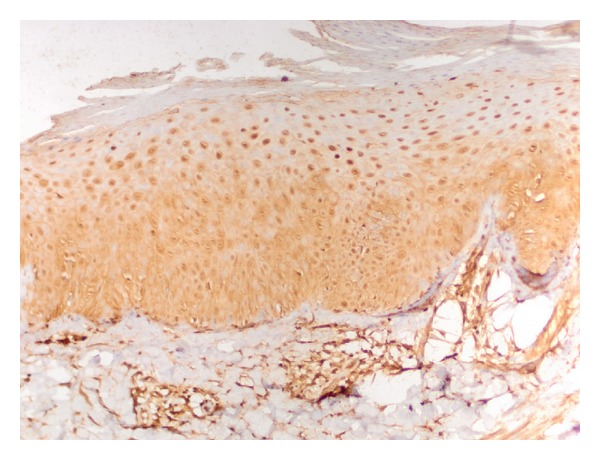
Diffuse nuclear and basal cytoplasmic staining with NF-kappa B in psoriatic epidermis (×200).

**Figure 4:**
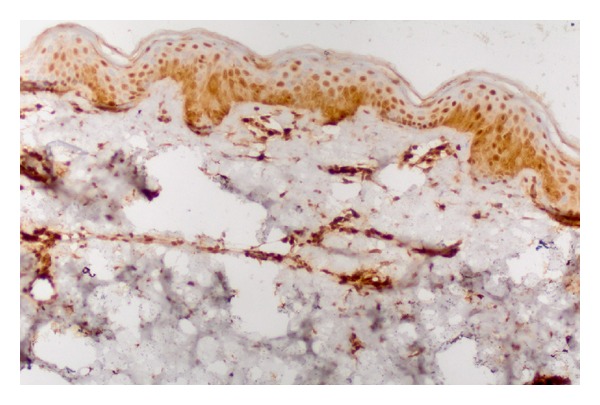
Diffuse nuclear and basal cytoplasmic staining with NF-kappa B in normal epidermis (×200).

Staining properties of NF-*κ*B are summarized in Table 2. Diffuse epidermal staining was seen in 10 psoriatic lesions: 1 cytoplasmic, 2 nuclear, and 7 nuclear + cytoplasmic. Superficial nuclear and basal cytoplasmic staining was seen in 20 psoriatic samples. Eleven psoriatic samples showed diffuse nuclear and basal cytoplasmic staining pattern (Figure 3). Eighteen normal skin samples showed diffuse nuclear and basal cytoplasmic staining. Superficial nuclear and basal cytoplasmic staining was seen in 3 normal skin samples (Figure 4).

## 4. Discussion

Survivin, one of the recently discovered IAP, is expressed in keratinocytic neoplasms and hyperproliferative lesions. Bowen et al. reported that survivin was expressed in most cases of actinic keratosis (83%), squamous cell carcinoma (100%), verruca vulgaris (91%), seborrheic keratosis (100%), and psoriasis (88%). Staining in psoriasis tended to be weaker and, in some cases, localized to the superficial (upper one-third of the epidermis) component of the lesions [2]. Markham et al. reported strong nuclear survivin expression in the basal layer of epidermis in 16 patients with psoriasis that decreased significantly with infliximab therapy [7]. Simonetti et al. found nuclear survivin expression mainly in the psoriatic suprabasal layer, while cytoplasmic survivin expression was seen in both the basal and suprabasal keratinocytes [8]. In normal skin, epidermal survivin expression was mostly negative, but they reported faint signals in the basal layer.

Indeed, growing evidence indicates that survivin is expressed in normal adult cells, particularly primitive hematopoietic cells, T lymphocytes, polymorphonuclear neutrophils, and vascular endothelial cells, and may regulate their proliferation or survival [9]. In Western blots, the antisurvivin antibody recognized a single band of 16.5 kDa in protein extracts from normal human keratinocytes in culture, in agreement with the predicted size of survivin [10].

NF-*κ*B activation is suggested to be necessary to either cause or maintain cell cycle arrest as keratinocytes commit to terminally differentiate and exit the basal layer. In this regard, the behavior of the epidermis seems to be opposite from that of the immune cells, where NF-*κ*B activity seems to play a positive role in proliferation. NF-*κ*B has been associated with promotion of apoptosis in double positive thymocytes, and, although controversial, NF-*κ*B activation may promote cell death in some neurons exposed to ischemia, or oxidative stress [4]. Li et al. reported that NF-*κ*B acts as a key bridge which links the activated Th1 cells with the transcription of many crucial genes involved in the pathogenesis of psoriasis, such as TNF-*α*, IL-8, IL-12, and cyclin D, and suggested that the dysfunction of NF-*κ*B may contribute to the formation or aggravation of psoriasis [11].

Doger et al. found significantly higher reactivity of NF-*κ*B in psoriatic epidermis, but NF-*κ*B positive cells located in different layers of psoriatic epidermis did not show a significant deviation from that of normal epidermis [12]. The authors suggested that in the setting of a chronic inflammatory state in psoriasis there is an imbalance between the antiapoptotic role and the cell cycle inhibitory role of NF-*κ*B, and in cells where NF-*κ*B is induced by TNF-*α* apoptosis may not occur leading to increased epidermal thickness and hyperproliferation.

Abdou and Hanout investigated survivin and NF-*κ*B expressions in psoriasis and found that survivin was expressed in 22 (73%) of 30 psoriasis samples either in epidermis, in endothelial cells of proliferating capillaries, or in both of them [6]. Twenty psoriasis cases (66%) showed epidermal nucleocytoplasmic positivity of NF-*κ*B, while control specimens showed only epidermal diffuse cytoplasmic staining. With these findings, the authors concluded that survivin and NF-*κ*B might be important factors in the pathogenesis of psoriasis.

In the present study, nuclear survivin expression throughout the epidermis was predominant in psoriasis. NF-*κ*B expression in different epidermal locations was seen in all of the psoriatic samples. The most common staining pattern (in 20 cases) was nuclear staining in superficial epidermal layers and cytoplasmic staining in the basal layer of the epidermis. Similar survivin and NF-*κ*B expressions were observed in normal skin samples; therefore, it is concluded that survivin and NF-*κ*B do not play an important role in epidermal proliferation and thickness seen in psoriasis.

## Figures and Tables

**Table 1:** Survivin expression.

	N*	N* + C*
Psoriasis	40	1
Normal skin	21	—

*Diffuse staining throughout the epidermis, N: nuclear, and C: cytoplasmic.

**Table 2:** NF-*κ*B Expression.

	Diffuse staining throughout the epidermis			DN + BC	SN + BC
	N	C	N + C
Psoriasis	2	1	7	11	20
Normal skin	—	—	—	18	3

N: nuclear, C: cytoplasmic, DN + BC: diffuse nuclear staining throughout the epidermis and cytoplasmic staining in the basal layer of the epidermis, and SN + BC: nuclear staining in superficial epidermal layers and cytoplasmic staining in the basal layer of the epidermis.
